# The impact of the Covid-19 pandemic on care of women with ectopic pregnancy in a tertiary London hospital

**DOI:** 10.52054/FVVO.13.4.042

**Published:** 2021-12-30

**Authors:** J.E. Gaughran, D.M. Geddes-Barton, T Cliff, F Bailey, C Ovadia, T Holland

**Affiliations:** Guy’s and St. Thomas’ Hospital Trust, Department of Obstetrics and Gynaecology; Kings College London Medical School, Department of Women and Children’s Health

**Keywords:** ectopic, covid, pandemic, laparoscopy, early pregnancy, tubal

## Abstract

**Background:**

In response to the COVID-19 pandemic, a central London tertiary referral hospital’s nurse-led Early Pregnancy & Acute Gynaecology Unit (EPAGU) suspended its walk-in service in favour of a telephone triage system with scheduled appointments.

**Objective:**

To assess if the pandemic and this adaptation to clinical services had an impact on the presentation, management and complication rate of ectopic pregnancies.

**Materials and Methods:**

A retrospective review was performed of ectopic pregnancies diagnosed in the EPAGU between 5th of March 2020 – 15th of July 2020 (pandemic) and 5th of March 2019 – 15th of July 2019 (pre- pandemic).

**Main outcome measures:**

Ultrasound findings, patient demographics, serum hCG concentrations, operative findings and complications.

**Results:**

There was a 36% reduction in attendances to the unit during the pandemic. Allowing for this, there was no significant difference in the diagnosis rate between the two periods. There was no significant difference in the gestation at diagnosis, serum hCG concentration or volume of mass at presentation. There was also no significant difference in rate of surgical intervention or complications including rupture of fallopian tube, haemoperitoneum or need for blood transfusion.

**Conclusion:**

This study suggests this is a safe means of caring for women with ectopic pregnancies which does not limit management options nor lead to higher complication rates.

**What is new:**

Other EPAGUs may choose to adopt a telephone triage system with reassurance of its safety.

## Introduction

On the 30^th^ of January 2020 the World Health Organisation (WHO) declared COVID-19 a public health emergency of international concern (WHO, 2019). A central London tertiary referral hospital issued a directive that departments should look at safe ways of reducing unnecessary travel to, and time spent in hospital and facilitate social distancing inside the building. Additionally, the International Society of Ultrasound in Obstetrics & Gynecology suggested in their guidance on COVID- 19 in pregnancy, that triage of patients should occur outside the outpatient and inpatient areas to reduce risk to patients and staff (Poon et al., 2020). Prior to this, our nurse-led Early Pregnancy and Acute Gynaecology Unit (EPAGU) offered a patient self- referral walk in service and open access for both general practitioners and Emergency Departments to send patients without referral. In response to the pandemic, both access points were suspended in favour of a nurse-led telephone triage system. During this telephone consultation, the patient’s history and self-reported signs and symptoms were discussed and electronically documented and a scheduled clinical and/or ultrasound appointment booked. A gynaecology team remained on call 24 hours a day to review any woman presenting unwell to the Emergency Department as per National Institute of Heath & Care Excellence (NICE) guidelines (NICE, 2014). The aim of this study was to assess if the COVID-19 pandemic and this adaptation to clinical services had an impact on the number of ectopic pregnancies diagnosed, the gestational age at diagnosis, the rupture rate, and suitable management options. A secondary aim was to assess the nursing team’s perception of the safety and acceptability of this alternative system.

## Methods

A retrospective review was performed of ectopic pregnancies diagnosed in the EPAGU of a central London tertiary referral hospital between 5^th^ of March 2020 – 15^th^ of July 2020 (pandemic) and 5^th^ of March 2019 – 15^th^ of July 2019 (pre- pandemic). Cases were identified by performing a search of the ultrasound database Astraia© (GMBH, Germany). The software’s audit function was used to search for the word ‘ectopic’ in both the diagnosis tab and also the free text box of reports produced during these two time points. Excluded cases were follow up ultrasound scans confirming the ongoing presence of an ectopic pregnancy diagnosed prior to the study dates as well as pregnancies of unknown location (due to these being a discrete cohort whose follow up and management options differ considerably to ectopic pregnancies). All scans were performed by either a sonographer or nurse sonographer with more than 5 years’ experience. All diagnoses were confirmed by either a Senior Fellow or Consultant Gynaecologist. In this unit, all patients have a serum Human Chorionic Gonadotropin (hCG) level measured at time of diagnosis. Blood results, operative notes and discharge summaries were reviewed on the hospital’s Electronic Patient Record. The following information was recorded: gestation at diagnosis (based on last menstrual period [LMP]); volume of ectopic mass measured on ultrasound; hCG, the number of patients opting for expectant; medical or surgical management; the need for a blood transfusion; if surgical management was employed, how many had an estimated blood loss (EBL) of more than 500ml and/or documentation of a ‘ruptured fallopian tube’. GraphPad Prism version 9.0 (GraphPad Software, USA) was used for statistical analysis. As the gestational age, mass volume and HCG values were non-normally distributed they were analysed using Mann- Whitney test with a two-tailed comparison. Median and Inter Quartile Ranges (IQR) are presented for this reason. For all other outcomes Fisher’s exact test was used. An email questionnaire containing the following 3 questions was distributed to the 13 nurses, 4 nursing assistants and 2 administrators working in the EPAGU:

1. Do you think a telephone triage service is a safe way of running the EPAGU?
2. Do you think we should continue a telephone triage service or return to a walk-in model?
3. Do you think a telephone service permits you more time to focus on patients in most need of urgent care?

The joint UK Medical Research Council & NHS Health Research Authority online decision making tool was used to determine there was no need for formal ethical approval as the data had already been collected as part of routine care; all care was complete; the data was anonymised and analysed within the direct clinical team (MRC & HRA, 2020). This decision was also supported by the hospital’s Research & Development Department.

## Results

**Figure 1:**
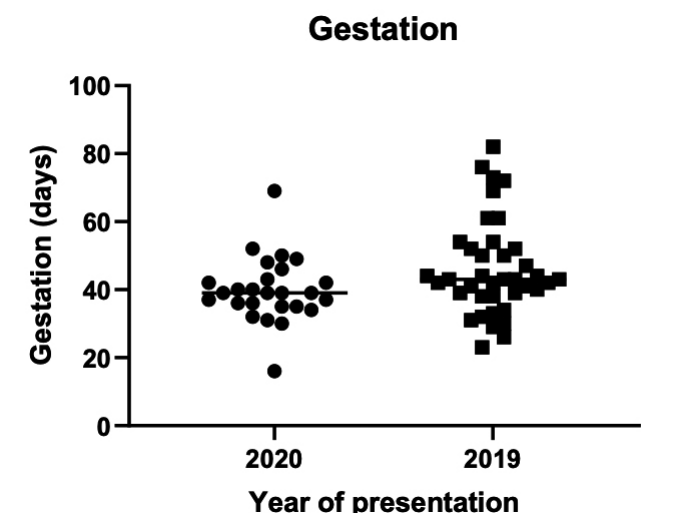
Gestation by LMP at time of diagnosis.

Between 5^th^ of March 2020 – 15^th^ of July 2020 (pandemic), 1463 pregnant patients presented to the EPAGU with either pain and/or vaginal bleeding, compared to 2254 between 5^th^ of March 2019 – 15^th^ of July 2019 (pre-pandemic): a 36% reduction. During the pandemic period, 31 ectopic pregnancies were diagnosed versus 42 the year preceding: a 27% reduction. Allowing for decreased attendances, there was no statistical significance in ectopic pregnancy diagnosis rate between the two periods. No patients transferred their care to another unit after receiving the diagnosis of ectopic pregnancy. In 2020, the median gestation at diagnosis was 39 days (IQR 34-43.75) compared to 43 days (IQR 38- 52) in 2019. There was no statistically significant difference (p= 0.4862) (Figure 1).

**Figure 2:**
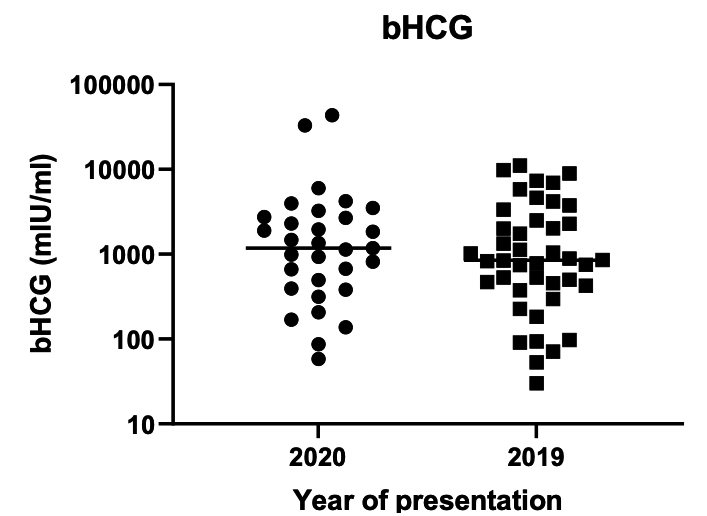
Median hCG at time of diagnosis.

Median HCG at diagnosis in 2020 was 1176mIU/ ml (IQR 395-2746) compared to 849mIU/ml (IQR 412-2719) in 2019 (p=1.0000) (Figure 2).

**Figure 3:**
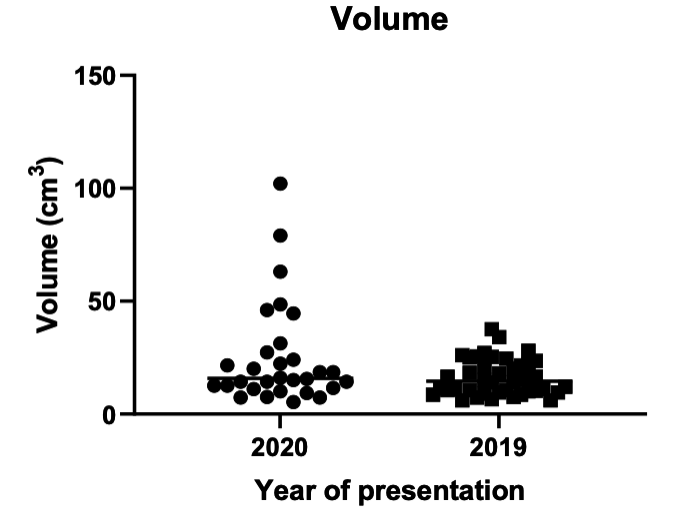
Volume of ectopic mass on ultrasound.

The volume of the mass during the pandemic did not significantly differ at 15.80cm^3^ (IQR 11.45- 28.30) compared to 14.40cm^3^ (IQR 9.90-37.60) pre-pandemic (p 0.6741) (Figure 3).

Management during the pandemic compared to the year preceding was as following: expectant management 11/31 (35%) versus 16/42 (38%) [OR =0.89 (0.34 to 2.32)]; medical management 7/31 (23%) versus 8/42 (19%) [OR =1.24 (0.42 to 3.58)]; surgical management 13/31 (42%) versus 18/42 (43%) [0.96 (0.37 to 2.40)]. There was no significant difference in any of the groups. In 2020, haemoperitoneum was documented in 14/31 (45%) of surgical cases compared to 12/42 (23%) in 2019 (OR= 2.06 [0.77 to 5.22], p= 0.6741). A ruptured fallopian tube was documented in 5/31 (16%) of cases during the pandemic compared to 8/42 (19%) pre pandemic (OR= 0.82 [0.26 to 2.55], p= 1.00). Estimated blood loss (EBL) was greater than 500ml in no cases in 2020 compared to 3/42 (7%) of cases in 2019 (OR= 0 [0.00 to 1.54], p= 0.67). Blood transfusion was given in no cases during the pandemic and in 1 case in 2019 (OR= 0.67, p= 1.00).

Of the 19 staff members sent questionnaires, 15 (79%) responded. Of the 15 respondents, 100% felt that the telephone triage system was a safe means of caring for women with complications in early pregnancy; 100% would favour continuing this rather than reverting to a walk-in service and 14/15 (93%) of staff agreed that cessation of walk-in services permitted more time to focus on patients most urgently in need of care.

## Discussion

To our knowledge, this is the first study to assess the safety of running a nurse-led EPAGU service via telephone triage rather than a walk-in service in response to the COVID-19 pandemic.

During the first peak of the COVID-19 pandemic there were 11 fewer ectopic pregnancies diagnosed compared to the preceding year. This is most likely explained by the reduced attendances to the unit. The unit geographically serves central London office workers and women commuting through one of the country’s busiest train stations. With a move to working from home, the volume of attendances would inevitably be lower. Additionally, emerging evidence suggests a reluctance of patients to attend hospital due to fears of contracting COVID-19 and due to hospital’s restrictions on visitors (ONS, 2020; Gaughran et al., 2020). The Office of National Statistics recorded a lower birth rate in 2020 compared to 2019 (ONS, 2020). Epidemiological studies have also demonstrated significant decline in birth rates during previous pandemics (Bloom- Feshbach et al., 2011). It is possible that fewer women attended our unit out of choice due to the cessation of walk in services, however there has been no published data to suggest an increase in number of attendances at other London EPAGUs to support this.

NICE mandate that all women should be seen in an EPAGU within 24 hours of developing symptoms that could be associated with ectopic pregnancy or miscarriage (NICE, 2014). Concerns were voiced by members of staff that changing the access pathway to the unit might cause confusion, which, alongside a reluctance to attend hospital, may lead to later presentations of ectopic pregnancies with associated morbidity. Our data did not support this. The Royal College of Obstetricians & Gynaecologist (RCOG) guideline on the management of ectopic pregnancy is widely accepted in the UK (Elson et al., 2016). This guideline advise that expectant management should only be advised if initial hCG levels are less than 1500IU/L and medical management can be offered if hCG is less than 5000IU/L and the mass less than 35mm with no signs of haemorrhage or fetal heartbeat. This study found no difference in hCG level at presentation or mass volume. In those managed surgically during the pandemic period the rates of haemoperitoneum were 45% vs 23% in the pre-pandemic period, however this was not statistically significant. Also, there was no increased rate of EBL >500ml or blood transfusion requirement in the pandemic period. This not only suggests a telephone triage system is safe, but also does not limit patients’ management options. One multicentre observational study in the UK found there was a significantly higher rate of non-surgical management of ectopic pregnancies during the first wave of the COVID-19 pandemic with no difference in complication rate (Platts et al., 2020). While our study did not find a significant difference in management choices, the lack of difference in complication rates in both studies further supports the safety of less invasive management options.

In May 2020 the RCOG published a guideline titled ‘Guidelines for rationalising early pregnancy services in the evolving coronavirus (COVID- 19) pandemic. This guideline supports the use of telephone triage (RCOG, 2020). Additionally, a joint RCOG and British Society for Gynaecological Endoscopy (BSGE) guidance recommended that non-surgical methods of treatment should be considered to reduce the risk of COVID-19 transmission to health care professionals and patients, provided they are safe. This study suggests that a telephone triage facilitates this without compromise to patient care (RCOG & BSGE, 2020).

The response from the EPAGU staff suggests that this approach is perceived to be both safe and desirable for working conditions.

Limitations of this study are the fact that it is retrospective and a relatively small sample size. With results suggesting this approach to be safe, our plan is to continue collecting larger volumes of data prospectively. Using the ultrasound software to identify patients for this study means that patients who potentially transferred their care to another hospital after telephone triage, but before a scan were not included. It was not feasible to contact other units to follow up on such patients. The questionnaire to staff was not anonymous which may have reduced response rates or introduced bias. Additionally, patients, general practitioners and Emergency Physicians’ opinions on this change of service was not assessed, nor the potential impact on patient flow through the Emergency Department. This will be assessed in a future prospective study. In conclusion, this study suggests that a nurse- led telephone triage service is as safe as a walk-in service in the management of ectopic pregnancy, and may be adopted by other units looking to safely manage patient flow through their unit and hospital both during the pandemic and beyond.
